# Design of Low-RCS Broadband High-Gain Antennas Based on Transmission Array Metasurface

**DOI:** 10.3390/mi13101614

**Published:** 2022-09-27

**Authors:** Jia Lu, Xiangyu Cao, Lili Cong, Kefeng Ji, Kun Gao

**Affiliations:** Information and Navigation College, Air Force Engineering University, Xi’an 710077, China

**Keywords:** metasurface, low RCS, broadband, high-gain

## Abstract

In this paper, a low-RCS broadband high-gain antenna based on metasurface transmission array is proposed, consisting of two parts: a metasurface transmission array and a feed antenna. When designing the metasurface transmission array, the phase compensation method is used to achieve the beam convergence effect of metasurface in the broadband. By designing the elements and using the checkerboard arrangement, the RCS of the incident wave with fixed polarization can be reduced more than 10 dB at X band or Ku band. The feed antenna is designed as a microstrip magnetic and electric dipole antenna, which has the characteristics of small structure and wide impedance bandwidth. An antenna that can reduce RCS by more than 10 dB in Ku band is simulated and measured. The measurement and simulation results show that the −10 dB operating bandwidth of the high-gain antenna designed in this paper is 6.7~13.5 GHz, and the relative bandwidth is 67%. The designed metasurface can effectively improve the gain of the antenna in the operating frequency band. In this way, the design of high-gain antenna is realized, and the antenna has an obvious RCS reduction effect on the vertically incident *y*-polarized wave in the whole Ku band. The method to design an antenna in this paper realizes the regulation of radiation and scattering at the same time, which has important reference significance for expanding the function of transmission array antennae and has great application value.

## 1. Introduction

High-gain antennas can effectively improve the performance of wireless communication systems and are widely used in civilian society and the military. Traditional high-gain antennas are mainly realized by an antenna array, reflector antenna, lens antenna and other methods. However, these methods have their own disadvantages in practical application. When using antenna arrays, there are some problems, such as complex feed structure and mutual coupling between elements. When using reflector antennas, there are some problems, such as transmission being blocked by feed and their high profile. Therefore, it is urgent to find a new designing method for high-gain antennas to break through these limitations.

Since Metasurface was proposed as a two-dimensional form of metamaterial, more and more examples have proved that the polarization, phase, amplitude and other characteristics of electromagnetic(EM) waves can be effectively regulated by designing the structure and arrangement of metasurface elements [1,2,3]. With the development of metasurface, using metasurface to design antennas has become a hotspot of research in recent years, which provides a new idea for the designing method of high-gain antennas [4,5,6,7,8,9,10,11]. For example, frequency selective surface (FSS) has been used to design high-gain antennas. Previous works [12,13] have studied the effect of FSS on antenna gain and bandwidth when they act as reflectors. Study [14] offers a viable prescription to enhance antenna gain for UWB applications by using FSS.

Transmission array antenna is one of the typical applications of metasurface for high-gain antennas. Based on the regulation of metasurface on phase, this kind of antenna can convert spherical wave into plane wave by the method of phase compensation, to achieve high gain. It has the advantages of simple feeding, high gain and not being blocked by feed [15,16,17,18,19,20]. Current studies on transmission array antennas mostly focus on the radiation characteristics [21,22,23,24,25], but there are some studies on reducing the Radar Cross Section (RCS). With the development of modern warfare, the characteristic of stealth has become an important index, and the stealth of the antenna is one of the important factors affecting the stealth efficiency of a carrier platform. Therefore, it is of great significance to design a transmission array antenna with both good radiation characteristics and low RCS characteristics to expand the function and application of transmission array antenna.

In this paper, an element with high transmission efficiency in a broadband is presented. Changing the size of the element can control the transmission phase of the EM waves in a wide band, and can control the reflection phase of a specific polarized incident wave at the same time. Based on this element, the metasurface transmission array is designed by using phase compensation and the checkerboard arrangement method, and a broadband magnetic and electric dipole feed is designed to combine with it. Finally, a broadband high-gain antenna with low RCS is designed based on the metasurface transmission array. An antenna that can reduce RCS by more than 10 dB in Ku band was processed and has been measured in a microwave anechoic chamber. The measurement results are in good agreement with the simulation results, which verify the effectiveness of the design and provide a good reference for the design of low-RCS broadband high-gain antenna.

## 2. Design and Simulation of Metasurface Element

### 2.1. Design of Element

X band has been widely used in civilian society and the military due to its strong penetration, small energy loss and strong anti-interference ability. Therefore, when designing a broadband antenna in this paper, the goal is to cover the whole X band at the operating frequency, which requires the metasurface to achieve a beam-focusing effect in the whole X band. When designing metasurface elements, the transmission amplitude of the elements should be as close as possible to 1, in order to reduce transmission loss. Moreover, in order to effectively convert the spherical wave radiated by the feed into the plane wave, it is necessary that the transmission phase of the element achieve 360° coverage.

**Figure 1:**
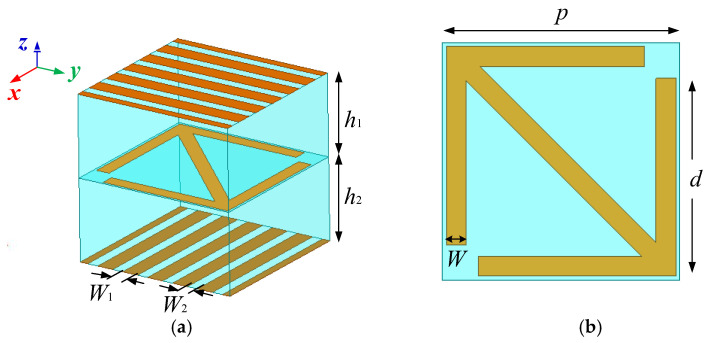
Structure diagram of metasurface element: (**a**) the element presented in this paper; (**b**) arrow-shaped metal patch.

An element that is similar to the Fabry–Perot resonant cavity and has high transmittance in the broadband was proposed in [26]. By changing the shape of the anisotropic structure in the middle layer, the phase of transmitted EM wave can be regulated. Inspired by this structure, this paper designs a metasurface element as shown in Figure 1a, in which the yellow part is metal and the blue part is a substrate layer. The upper and lower metal grids are arranged along the *y*-axis and *x*-axis respectively for polarization selection. The metal arrow in the middle is placed along the diagonal direction for polarization conversion. After simulation and optimization, the specific structural parameters of the element are *h*_1_ = *h*_2_ = 3 mm, *W*_1_ = 0.4 mm, *W*_2_ = 0.6 mm, *p* = 6 mm, and *W* = 0.5 mm. The relative permittivity is 2.65 and the dielectric loss tangent is 0.001.

**Figure 2:**
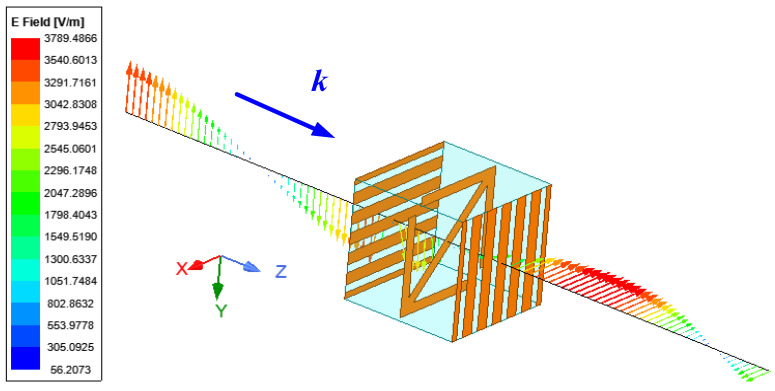
Electric field vector distribution on the central axis at 10 GHz.

**Figure 3:**
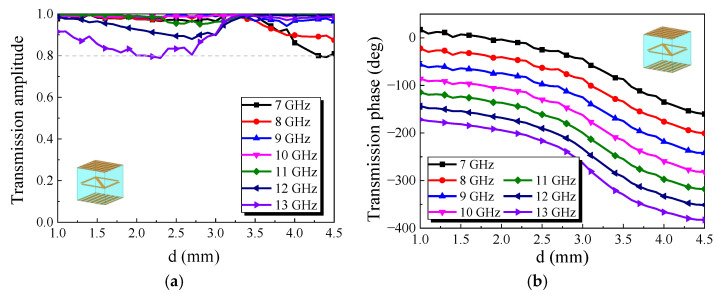
Simulation results of the element: (**a**) Transmission amplitude; (**b**) Transmission phase.

ANSYS HFSS software was used to simulate the element, as shown in Figure 2. The element can convert the *y*-polarized wave into an *x*-polarized wave. This element borrows the principle of the Fabry–Perot resonant cavity. There are EM waves parallel and orthogonal to the direction of the metal grid in the element. The orthogonal part will be transmitted out of the element, and the parallel part will be reflected by the metal grid. After the reflection and refraction, the element can achieve efficient transmission. Figure 3 show the simulation results of the element. It can be seen that when the *d* is changed, the element can maintain the transmission amplitude above 0.8 in the range of 7 GHz to 13 GHz, and the transmission phase changes stably. The variation in transmission phase can cover 180°s. An additional 180° phase can be obtained by rotating the middle metal arrow 90°s. Thus, 360° changes in phase are obtained.

According to the simulation results in Figure 3, the element has very good transmission performance in broadband. However, when the *y*-polarized wave is incident along the -*z* direction, it will be reflected by the transmission array. If using the element to design the transmission array antenna, the RCS of the antenna will be very high. This is detrimental to the antenna’s stealth. So, the element needs to be further optimized.

**Figure 4:**
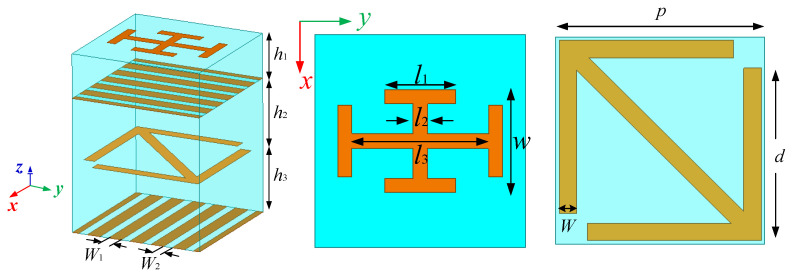
Structure diagram of metasurface element.

When designing low-RCS elements, the absorbing structure with resistance will make the element structure complicated. In comparison, it is simpler to design an element according to the principle of phase elimination and arrange them in a checkerboard structure. Since the element is designed with a metal grid structure, the top grid of the element is equivalent to a total reflective surface for incident waves that its polarization direction is parallel to the metal grid. So, the metal grid can act as the bottom surface. In order to control the reflection phase, a substrate layer and a cruciform metal patch are loaded on the base of the element in Figure 1, as shown in Figure 4. Because the cruciform patch is placed along the coordinate, it has no effect on the polarization conversion for EM wave, but mainly plays a role in adjusting the reflection phase of the *y*-polarized wave. The material with relative permittivity of 2.65 and dielectric loss tangent of 0.001 is also selected for the new substrate layer. The specific sizes of each layer are shown in Table 1.

### 2.2. Simulation and Analysis of Element

**Figure 5:**
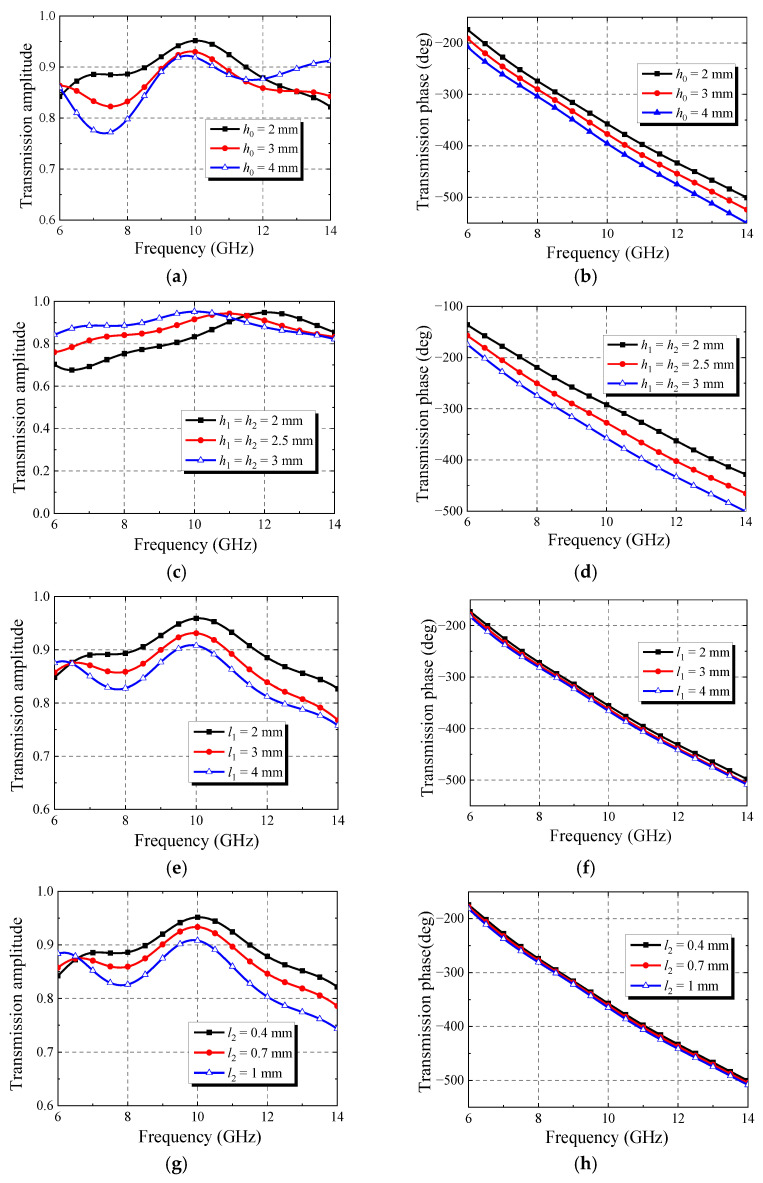

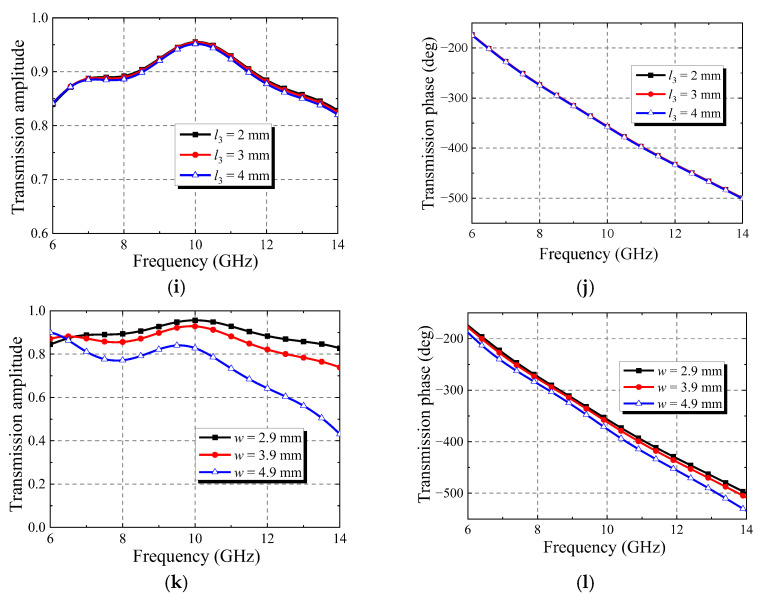
The effect of different parameters on the transmission performance of the element: (**a**) the effect of *h*_1_ on amplitude; (**b**) the effect of *h*_1_ on phase; (**c**) the effect of *h*_2_ on amplitude; (**d**) the effect of *h*_2_ on phase; (**e**) the effect of *l*_1_ on amplitude; (**f**) the effect of *l*_1_ on phase; (**g**) the effect of *l*_2_ on amplitude; (**h**) the effect of *l*_2_ on phase; (**i**) the effect of *l*_3_ on amplitude; (**j**) the effect of *l*_3_ on phase; (**k**) the effect of *w* on amplitude; (**l**) the effect of *w* on phase.

The designed metasurface element was simulated by HFSS. As shown in Figure 5, the effects of different parameters on the transmission performance of the element are simulated, respectively. The simulation results show that the transmission amplitude and the transmission phase of the element are almost unaffected by the change in parameter *l*_3_, but the transmission amplitude and the transmission phase of the element will be significantly affected when the other parameters increase. Therefore, *l*_3_ is taken as the variable in the design of this paper.

**Figure 6:**
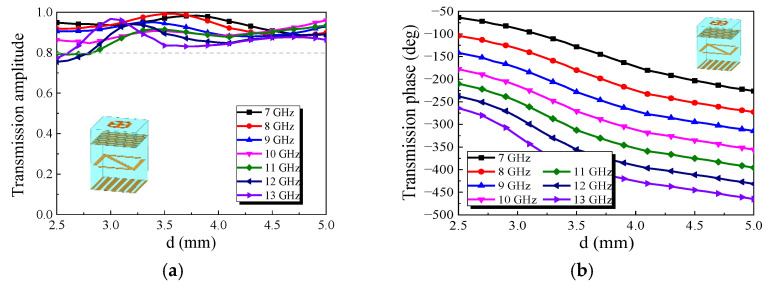

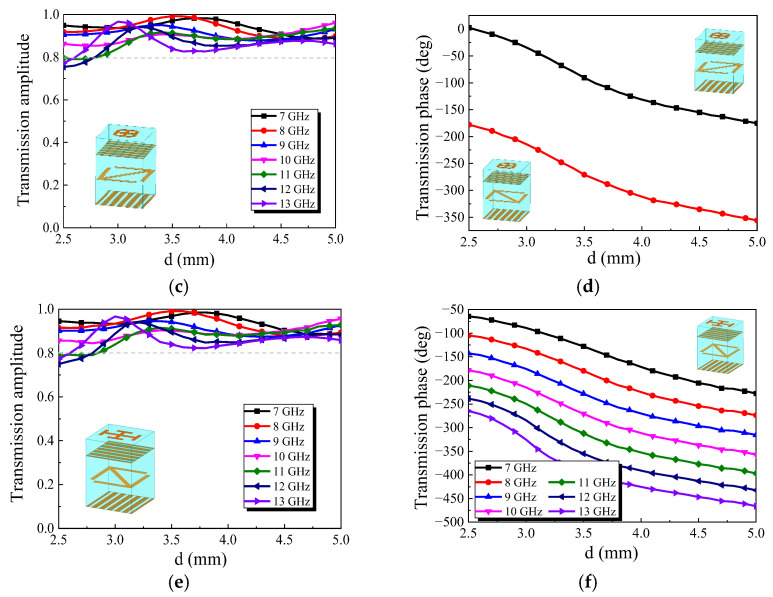
Simulation results of transmission performance: (**a**) transmission amplitude when *l*_3_ = 2 mm; (**b**) transmission phase when *l*_3_ = 2 mm; (**c**) transmission amplitude of the arrow rotated 90°; (**d**) phase comparison before and after arrow rotation at 10GHz; (**e**) transmission amplitude when *l*_3_ = 3.9 mm; (**f**) transmission phase when *l*_3_ = 3.9 mm.

The simulation results of transmission performance are shown in Figure 6. The value of *h*_1_ is set as *h*_1_ = 2 mm. Figure 6a is the simulation result of the transmission amplitude. Compared with Figure 3a, it can be seen that when *d* is changed in the range of 2.5–5 mm, the transmission amplitude of the element is decreased in the frequency range of 7–13 GHz, but it can still be kept above 0.8. Figure 6b shows the simulation results of the transmission phase of the element. When *d* is varied in the range of 2.5–5 mm, the transmission phase of the element can maintain stable phase variation in the frequency range of 7–13 GHz, and the change of phase can achieve a coverage of 180°s. It can be seen from Figure 6c,d that the transmission amplitude is basically unchanged after rotating the metal arrow by 90°s, but an additional 180°s of phase can be obtained. Given *l*_3_ = 3.9 mm and having run the simulation, the results are shown in Figure 6e,f. After comparing Figure 6e,f with Figure 6a,b, it can be seen that although a layer of substrate and metal patch are loaded, the element can maintain good transmission performance and can be used to design a broadband metasurface transmission array.

**Figure 7:**
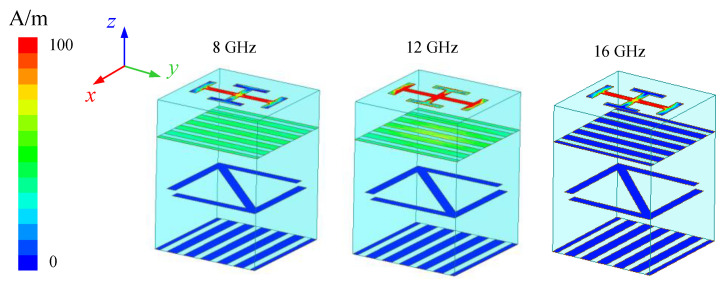
Surface current distribution of element when *y*-polarized wave incident.

As shown in Figure 7, it is the surface current distribution of the element when *y*-polarized EM wave is incident along the direction of −*z*. It can be seen from the figure that the induced current is only distributed in the first two layers of the element. It is indicated that the incident EM wave cannot through the upper metal grid to propagate. Therefore, by changing the length of *l*_3_, the reflected wave property of the element can be regulated. After optimizing the *l*_3_, the design of low RCS metasurface can be realized when the reflected phase meets the condition of phase cancellation.

At present, X-band and Ku-band are the most commonly used working frequencies for fire control radar, imaging radar and guidance radar. Therefore, it is more practical to effectively reduce the RCS on X-band or Ku band, which requires the reflection phase of the element to meet the condition of phase cancellation in these frequencies.

**Figure 8:**
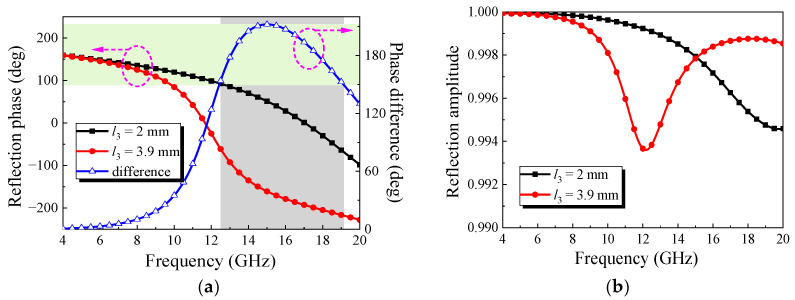
Simulation results of reflection performance: (**a**) reflection phase of the *y*-polarized incident wave; (**b**) reflection amplitude of the *y*-polarized incident wave.

Figure 8 shows the simulation results of the element’s reflection performance. When *l*_3_ = 2 mm and *l*_3_ = 3.9 mm, the reflection phase of *y*-polarized incident waves has a 180° phase difference in the range of 12.4–19.2 GHz, and the reflection amplitude is approximately 1. In the theory, the reduction value of RCS could be more than 10 dB in the Ku band by checkerboard arrangement of these two elements.

**Figure 9:**
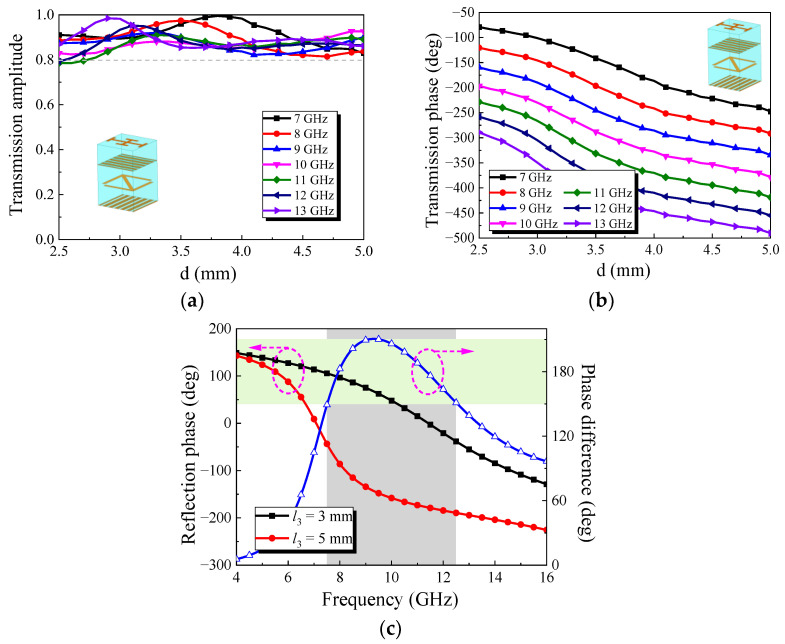
Simulation results of the element: (**a**) transmission amplitude; (**b**) transmission phase; (**c**) reflection phase of the *y*-polarized incident wave.

In general, it can cause the resonant frequency to shift to the lower frequency, increasing the thickness of the substrate layer. After the optimization of the top layer, the value of *h*_1_ is set as *h*_1_ = 3 mm, and the simulation results of the element are shown in Figure 9.

Figure 9a shows the simulation results of transmission amplitude. When *d* is varied in the range of 2.5–5 mm, the element can still maintain a transmission amplitude above 0.8 in the frequency range of 7–13 GHz. Figure 9b shows the simulation results of the transmission phase. When *d* is varied in the range of 2.5–5 mm, the transmission phase of the element can achieve 180° coverage in the range of 7–13 GHz. Figure 9c is the simulation result of the reflection phase. For a *y*-polarized wave incident along the direction of −*z*, it can be seen that the element could have a 180° phase difference in 7.5–12.5 GHz when *l*_3_ = 3 mm and *l*_3_ = 5 mm. A comprehensive analysis of the simulation results in Figure 9 shows that the element can theoretically cause the RCS reduction value to exceed 10 dB in the X band when *h*_1_ = 3 mm.

## 3. Design and Simulation of Metasurface Transmission Array Antenna

Through the above analysis, the proposed element can be used to construct the transmission array, and has been demonstrated to have a reduction effect of more than 10 dB RCS in X band or Ku band. The design methods and principles of the two kinds of reduction effect antennas are the same. This paper takes the antenna which RCS can reduce more than 10 dB in the Ku band as an example for design.

### 3.1. Design of Metasurface Transmission Array

In order to achieve the focusing effect of metasurface that can transform the spherical wave into the plane wave, the distribution of the phase should be satisfied as per Equation (1).

(1) $$\varphi \left(x,y\right)=k_{0}\left(\sqrt{x^{2}+y^{2}+f^{2}}-f\right)+\varphi_{0},$$

Because the phase of each element is discontinuous, the phase of the element (m, n) on the array can be expressed as Equation (2).

(2) $$\varphi \left(m,n\right)=k_{0}\left(\sqrt{{\left(mp\right)}^{2}+{\left(np\right)}^{2}+f^{2}}-f\right)+\varphi_{0},$$

**Figure 10:**
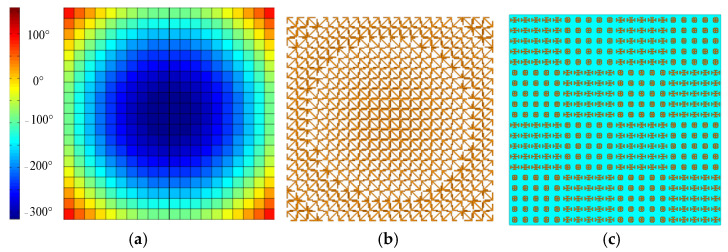
Structure diagram of metasurface transmission array: (**a**) phase distribution; (**b**) metal arrow corresponding to different phase; (**c**) checkerboard distribution.

$k_{0}=2\pi /\lambda_{0}$, *f* is the focal length, and $\varphi_{0\;}$is the phase of the central position element on the metasurface. After optimization, the array size is set as 20 × 20, the focal diameter ratio of the metasurface transmission array is set as 0.5, and the center frequency is set as 10GHz. By calculation, the phase distribution of the designed metasurface transmission array is shown in Figure 10a, and the size distribution of the corresponding metal arrow is shown in Figure 10b. The elements are composed into a 5 × 5 subarray, and then the subarray is arranged in a checkerboard pattern of 4 × 4 to achieve the low RCS design of the metasurface transmission array, as shown in Figure 10c. The blue part is the substrate layer and the yellow part is the metal patch.

### 3.2. Design of Feed

In order to realize the broadband of the antenna, in addition to designing a metasurface with good transmission performance in the broadband, a broadband feed antenna should also be designed. A microstrip magnetoelectric dipole antenna was proposed in [27], which replaced the vertical metal plate in the magnetoelectric dipole antenna with metal via a hole. It has the characteristics of small structure and wide impedance bandwidth, and provides ideas for the design of a feed antenna in this paper.

**Figure 11:**
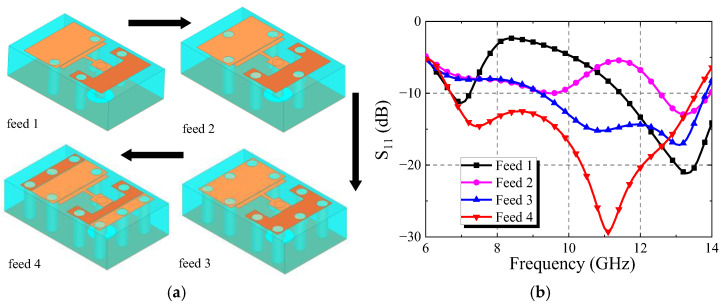
Simulation results of feed antenna (**a**) Design process of feed; (**b**) S_11_ curve.

**Figure 12:**
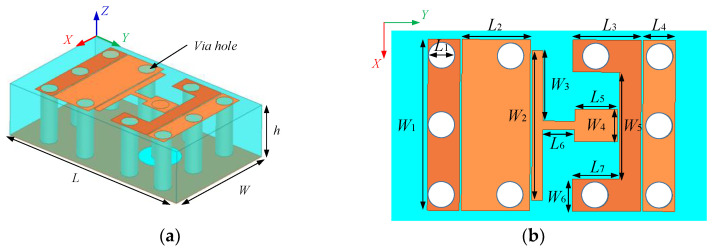
Structure diagram of feed antenna: (**a**) three-dimensional structure diagram; (**b**) structure diagram of radiation patch.

Figure 11 shows the design flow of broadband feed antenna and its S_11_ curve. The structure of feed 1 is similar to that in literature [27], its resonant points are 7 GHz and 13 GHz, but the matching performance on the X-band is poor. A metal via hole is punched symmetrically at both ends of feed 1 to form feed 2. It can be seen that the S_11_ of feed 2 in the intermediate frequency decreases somewhat, and the resonant point moves to high frequency. Feed 3 is formed by adding two metal pieces via holes at each end of feed 2. It can be seen that the antenna has good matching performance in 9.5–13.8 GHz, but has no obvious effect on the frequency band below 9.5 GHz. In order to improve the matching of the low frequency and expand the impedance bandwidth of the antenna, the radiating patch was slotted symmetrically to form feed 4. It can be seen from the simulation results that the resonant point of the feed source moves to the lower frequency after slotting, and the bandwidth meets the design requirements. After optimization, a feed antenna operating from 6.7 GHz to 13.5 GHz has been successfully designed. The structure of the feed antenna is shown in Figure 12. The blue part is the substrate layer with relative permittivity of 2.65, and the yellow part is the metal patch. Table 2 shows the specific size of the antenna.

**Figure 13:**
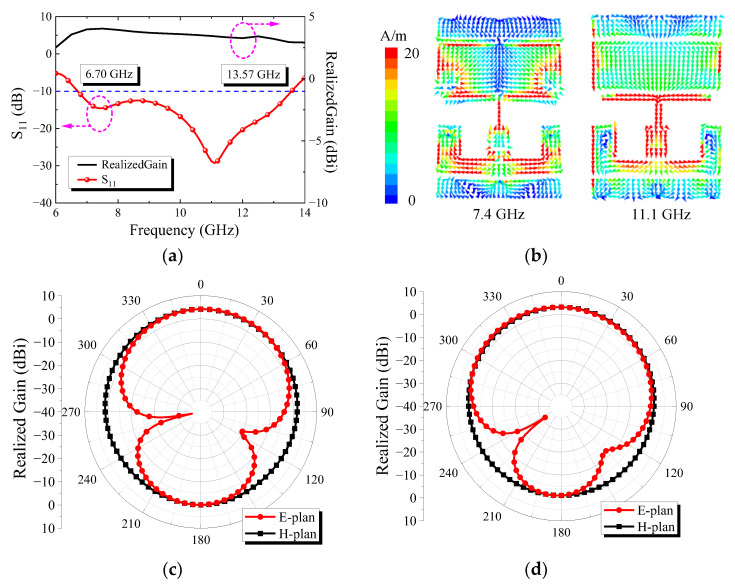
Simulation results of feed radiation performance: (**a**) curve of S_11_ and realized gain; (**b**) surface current distribution at 7.4 GHz and 11.1 GHz; (**c**) radiation pattern at 7.4 GHz; (**d**) radiation pattern at 11.1 GHz.

Figure 13a shows that the antenna exhibits resonant points at 7.4 GHz and 11.1 GHz. Figure 13b shows the surface currents’ distribution at 7.4 GHz and 11.1 GHz. It can be seen that the current distributions at these two resonant points are different, indicating that there are two different resonant modes, so the antenna can work with broadband. Moreover, the surface current of the antenna flows along the y-axis, so the polarization direction of the antenna is y-polarization. Figure 13c,d show the radiation pattern at 7.4 GHz and 11.1 GHz. It can be seen that the patterns on the E-plane and H-plane are symmetrical, but the E-plane is not as symmetrical as the H-plane. This is because the structure of the antenna is symmetric about the y-axis, resulting in the current distribution also being symmetric about the y-axis, so the radiation pattern is more symmetric on the H-plane.

### 3.3. Ultra-Surface Antenna Design and Simulation

**Figure 14:**
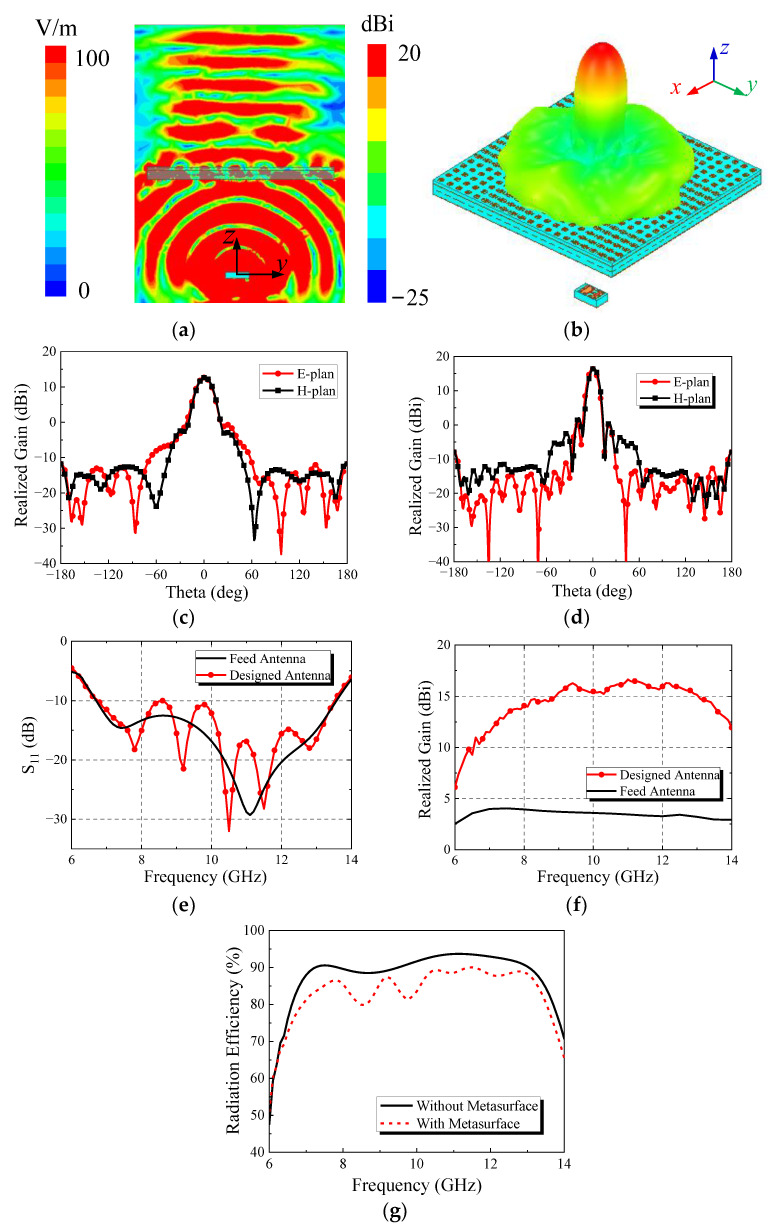
Simulation results of antenna: (**a**) electric field distribution of yoz plane at 10 GHz; (**b**) three-dimensional far-field pattern at 10 GHz; (**c**) radiation pattern at 7.4 GHz; (**d**) radiation pattern at 11.1 GHz; (**e**) S_11_ curve; (**f**) maximum radiation direction gain; (**g**) radiation efficiency.

By placing the feed antenna at the focal point of the metasurface transmission array, the desired high-gain antenna system can be formed. Figure 14 shows the simulation results of the antenna’s radiation performance. It can be seen from the figure that the spherical wave radiated by the feed antenna is effectively transformed into plane wave by the metasurface, which causes the antenna radiation to become more concentrated and thus to have the characteristic of high gain. The maximum gain is 16.6 dB, and the 3 dB bandwidth of gain is 57.8%. At the same time, the antenna maintains good broadband characteristics and realizes the design purpose for the working bandwidth to cover the X band. However, compared with the feed antenna, the resonant points are increased. 

**Figure 15:**
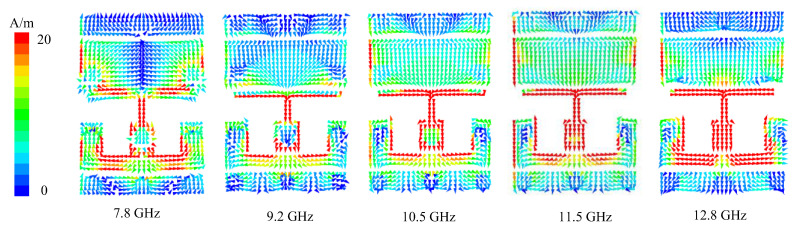
Surface current distribution at resonant point.

Figure 15 is the surface current distribution of the antenna at each resonant point. It can be seen from the figure that although the antenna has five resonant points, the current distribution at the four resonant points (9.2 GHz, 10.5 GHz, 11.5 GHz and 12.8 GHz) is similar and belongs to the same resonant mode. By comparing it with Figure 13b, it can be seen that the current resonance mode of the feed antenna is not changed after loading the metasurface, so the bandwidth of the transmission array antenna is basically the same as that of the feed. The increase of the resonant points is due to the change in the impedance matching.

**Figure 16:**
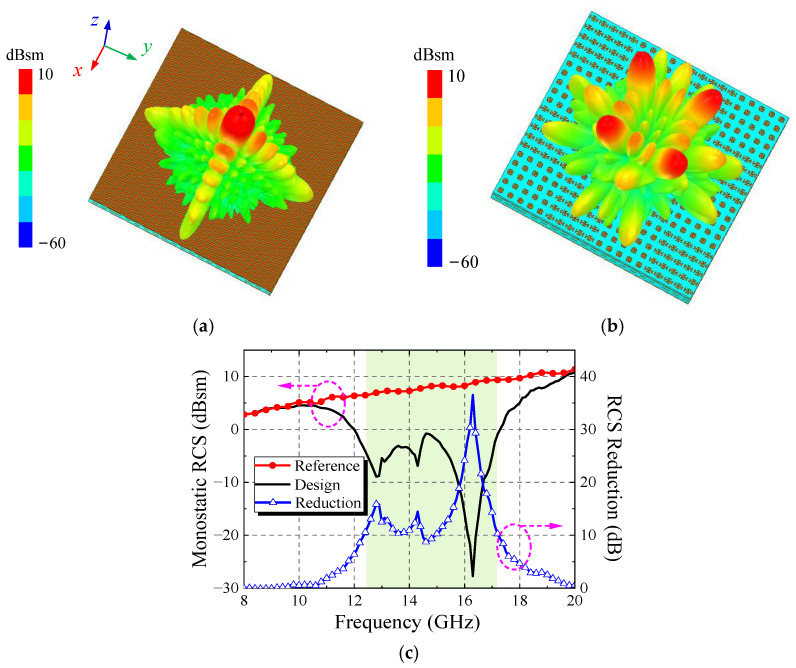
Simulation results of scattering performance on Ku band: (**a**) without cruciform patch; (**b**) checkerboard arrangement with cruciform patch; (**c**) RCS reduction effect in Ku band.

In order to analyze the scattering performance of the antenna, the *y*-polarized EM wave is set to incident on the surface of the transmission array antenna. The simulation results are shown in Figure 16. Compared with the transmission array antenna composed of elements without cruciform patch, the antenna has an obvious RCS reduction effect in the range of 11–19 GHz, and the RCS reduction in the range of 12.3–17.3 GHz can reach more than 10 dB. It shows that the proposed metasurface antenna achieves a good RCS reduction effect in Ku band.

**Figure 17:**
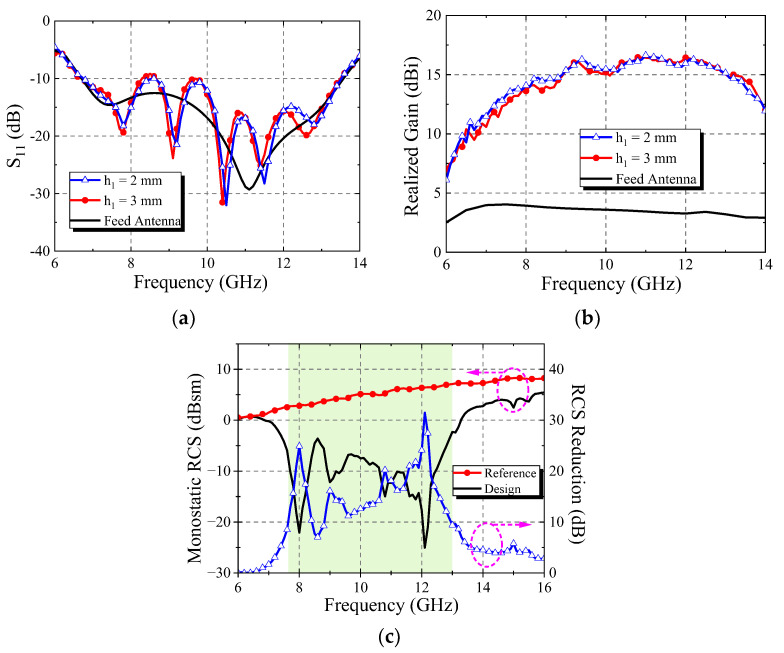
Simulation results of antenna: (**a**) S_11_ curve; (**b**) realized gain; (**c**) RCS reduction effect in X band.

The element with *h*_1_ = 3 mm was used for metasurface antenna design, and the same arrangement as Figure 16b was simulated. The simulation results are shown in Figure 17. It can be seen that the bandwidth is basically unchanged, the gain is basically the same, and the RCS reduction can exceed 10 dB in the range of 7.5–13 GHz. It indicates that the proposed metasurface antenna can maintain the original radiation characteristics. The RCS of the antenna can be reduced by more than 10 dB in the X-band to achieve a good low scattering effect.

## 4. Measurement and Result Analysis of Metasurface Antenna

**Figure 18:**
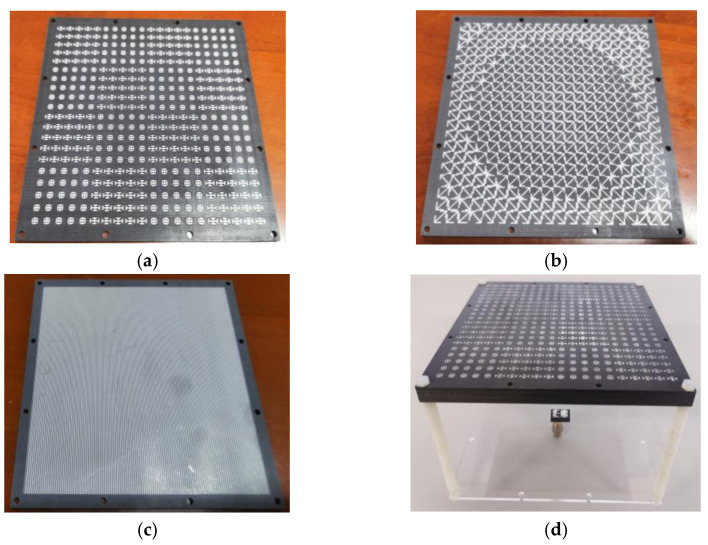
Finished product: (**a**) checkerboard structure; (**b**) metal arrow; (**c**) metal grid; (**d**) transmission array antenna.

As shown in Figure 18, in order to verify the simulation results, the transmission array antenna was processed. During processing, the metasurface was processed in three layers. Figure 18a is the checkerboard structure with metal grid on its back. Figure 18b shows the layer of the metal arrow. Figure 18c shows the bottom metal grid. In addition, the substrate layer of the metasurface was widened, and through-holes with a diameter of 3 mm were punched to facilitate the combination with the metasurface. As shown in Figure 18d, in order to facilitate the fixation of the feed antenna, the feed is fixed on a transparent acrylic plate. Four through-holes with diameters of 3 mm were punched on the acrylic plate. Four nylon screws were used to fix the antenna and strictly control the height of the air cavity to form a transmission array antenna.

**Figure 19:**
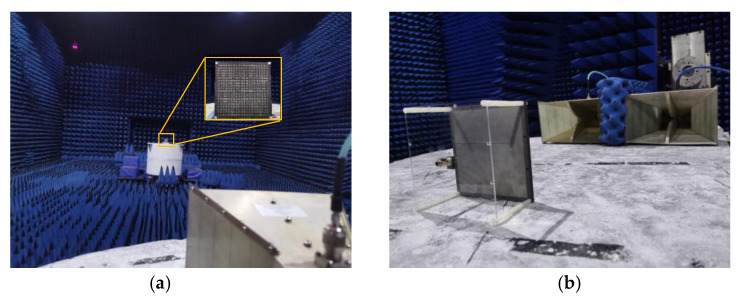
Measurement environment: (**a**) measurement of radiation performance; (**b**) measurement of scattering performance.

**Figure 20:**
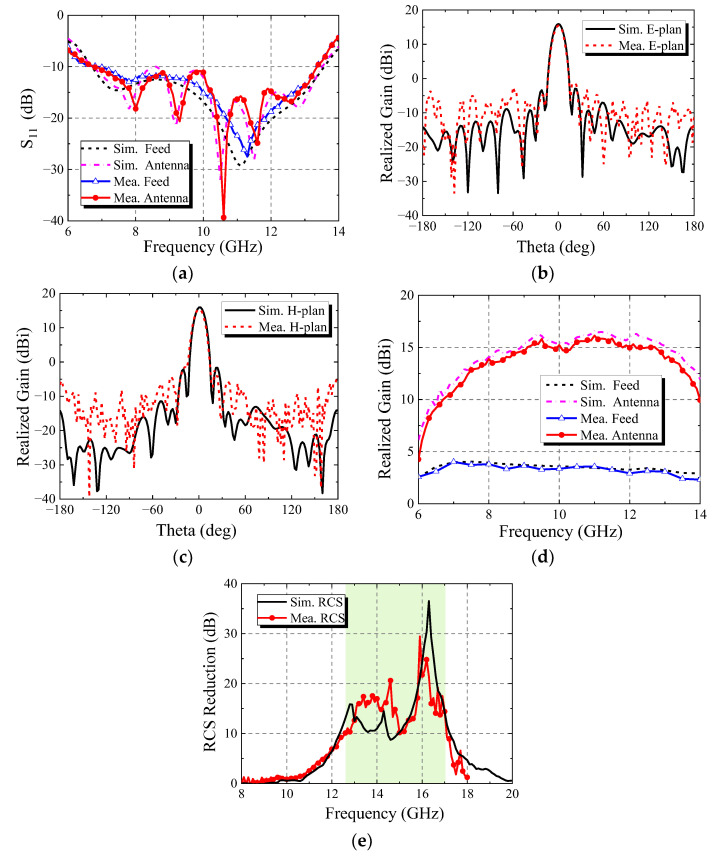
Comparison between simulation results and measurement results: (**a**) S_11_ curve; (**b**) radiation pattern of E-plan at 10.5 GHz; (**c**) radiation pattern of H-plan at 10.5 GHz; (**d**) maximum radiation direction gain; (**e**) RCS reduction.

The measurement environment of the anechoic microwave chamber is shown in Figure 19. An Agilent N5230C vector network analyzer was used to test the S_11_ curve of the antenna. The radiation performance and scattering performance of the antenna were measured in the microwave anechoic chamber. Figure 20 shows the measurement results of the antenna. From the comparison, it can be seen that there are some differences between the simulation results and the measurement results. This is mainly due to environmental and machining errors, and the difference is within the allowable range of error. The measured results verify the feasibility of the design in this paper.

## 5. Conclusions

In this paper, a low-RCS broadband high-gain antenna design method based on a metasurface transmission array is proposed. The working bandwidth of the antenna can cover the whole X-band. By adjusting the structure of the metasurface element, the designed antenna can effectively reduce the RCS in X band or Ku band. An antenna with RCS reduction greater than 10 dB in the Ku band is processed and measured. The gain is improved in the working frequency range of 6.7–13.5 GHz with a maximum increase of 12.7 dB. RCS reduction was achieved in the range of 10.5–18 GHz with a maximum reduction of 29.5 dB. The simulation results are in good agreement with the measurement results, which proves the effectiveness of the design. Compared with the traditional high-gain antenna, this version has obvious advantages, which demonstrate important reference significance and great potential application value.

## Figures and Tables

**Table 1:** Size of metasurface unit.

*p*	*h*_1_	*h*_2_	*h*_3_	*W*_1_	*W*_2_	*l*_1_	*l*_2_	*w*	*W*
6	2	3	3	0.4	0.6	2	0.4	2.9	0.5

**Table 2:** Size of feed antenna.

*L*	*L*_1_	*L*_2_	*L*_3_	*L*_4_	*L*_5_	*L*_6_	*L*_7_	*W*	*W*_1_	*W*_2_	*W*_3_	*W*_4_	*W*_5_	*W*_6_	*h*
15	1.2	3.2	3.2	1.5	2	1.5	2.2	9	8	7	3.3	1.5	5	1.5	4

## Data Availability

Not applicable.
